# High Frequency of the EMRSA-15 Clone (ST22-MRSA-IV) in Hospital Wastewater

**DOI:** 10.3390/microorganisms10010147

**Published:** 2022-01-11

**Authors:** Vanessa Silva, Jessica Ribeiro, Jaqueline Rocha, Célia M. Manaia, Adriana Silva, José Eduardo Pereira, Luís Maltez, José Luis Capelo, Gilberto Igrejas, Patrícia Poeta

**Affiliations:** 1 Microbiology and Antibiotic Resistance Team (MicroART), Department of Veterinary Sciences, University of Trás-os-Montes and Alto Douro (UTAD), 5000-801 Vila Real, Portugal; vanessasilva@utad.pt (V.S.); jessicalribeiro97@gmail.com (J.R.); adrianaa.silva95@gmail.com (A.S.); jeduardo@utad.pt (J.E.P.); lmaltez@utad.pt (L.M.); 2 Department of Genetics and Biotechnology, University of Trás-os-Montes and Alto Douro, 5000-801 Vila Real, Portugal; gigrejas@utad.pt; 3 Functional Genomics and Proteomics Unit, University of Trás-os-Montes and Alto Douro (UTAD), 5000-801 Vila Real, Portugal; 4 Associated Laboratory for Green Chemistry (LAQV-REQUIMTE), University NOVA of Lisboa, 1099-085 Lisboa, Caparica, Portugal; 5 CBQF—Centro de Biotecnologia e Química Fina—Laboratório Associado, Escola Superior de Biotecnologia, Universidade Católica Portuguesa, Rua Diogo Botelho 1327, 4169-005 Porto, Portugal; jrocha@ucp.pt (J.R.); cmanaia@ucp.pt (C.M.M.); 6 Veterinary and Animal Research Centre, Associate Laboratory for Animal and Veterinary Science (AL4AnimalS), University of Trás-os-Montes and Alto Douro (UTAD), 5000-801 Vila Real, Portugal; 7 BIOSCOPE Group, LAQV@REQUIMTE, Chemistry Department, Faculty of Science and Technology, NOVA University of Lisbon, 2825-466 Almada, Portugal; jlcm@fct.unl.pt; 8 Proteomass Scientific Society, Costa de Caparica, 2825-466 Setubal, Portugal

**Keywords:** *Staphylococcus aureus*, MRSA, EMRSA-15, hospital, wastewaters, effluents

## Abstract

Hospital wastewaters often carry multidrug-resistant bacteria and priority pathogens, such as methicillin-resistant *Staphylococcus aureus* (MRSA). Pathogens and antibiotic resistance genes present in wastewaters may reach the natural environment facilitating their spread. Thus, we aimed to isolate MRSA from wastewater of 3 hospitals located in the north of Portugal and to characterize the isolates regarding the antimicrobial resistance and genetic lineages. A total of 96 wastewater samples were collected over six months. The water was filtered, and the filtration membrane was immersed in BHI broth supplemented with 6.5% of NaCl and incubated. The inoculum was streaked in ORSAB agar plates for MRSA isolation. The isolates susceptibility testing was performed against 14 antimicrobial agents. The presence of resistance and virulence genes was accessed by PCR. Molecular typing was performed in all isolates. From the 96 samples, 28 (29.2%) were MRSA-positive. Most isolates had a multidrug-resistant profile and carried the *mec*A, *bla*Z, *aac*(6′)-Ie-*aph*(2″)-Ia, *aph*(3′)-IIIa, *erm*A, *erm*B, *erm*C, *tet*L, *tet*M, *dfr*A *dfr*G and *cat*_pC221_ genes. Most of the isolates were ascribed to the immune evasion cluster (IEC) type B. The isolates belonged to ST22-IV, ST8-IV and ST105-II and *spa*-types t747, t1302, t19963, t6966, t020, t008 and tOur study shows that MRSA can be found over time in hospital wastewater. The wastewater treatment processes can reduce the MRSA load. The great majority of the isolates belonged to ST22 and *spa*-type t747 which suggests the fitness of these genetic lineages in hospital effluents.

## 1. Introduction

Hospitals are ecological niches for antimicrobial resistant bacteria since up to one third of the hospitalized patients receive antibiotic therapy [1]. These bacteria may be spread by colonized patients, including through sewage and therefore hospital wastewaters [2]. Hospital effluents may be hazardous due to the presence of infectious agents and toxic substances, which include not only drugs and their metabolites, but also diagnostic agents, disinfectants, among other [3]. Antibiotics are one of such types of substances that are excreted mainly unmetabolized and once in wastewaters may contribute to the development of multidrug-resistant bacteria, mainly due to the fact that last resort antibiotics are often used in hospitals [4,5]. Antibiotics concentrations in hospital wastewaters are significantly lower than the therapeutic dosages. The sub-inhibitory concentrations present in wastewater are sufficient to induce a selective pressure on bacteria and are probably one of the drivers for resistance. Furthermore, it also favours the intraspecies and interspecies horizontal transfer of resistance genes [6,7]. In general, hospital wastewaters, classified as domestic effluents, are discharged into public sanitation networks, being treated in municipal Wastewater Treatment Plants (WWTPs) [3]. Thus, hospital wastewaters may represent major sources of pathogenic organisms in public wastewater, playing an important role regarding multidrug-resistant pathogens and also in the propagation of antibiotic resistance in the environment [7]. In fact, many studies have shown that hospital wastewaters contribute to the mobilization and dissemination of important nosocomial pathogens as well as antimicrobial resistance genes and genetic determinants which may reach surface waters, influencing the aquatic ecosystems and interfering with the food chain [8,9,10,11,12].

Methicillin-resistant *Staphylococcus aureus* (MRSA) belongs to the ESKAPE pathogens (*Enterococcus faecium*, *Staphylococcus aureus*, *Klebsiella pneumoniae*, *Acinetobacter baumannii*, *Pseudomonas aeruginosa*, and Enterobacter species) group that have the ability to “escape” from common antimicrobial treatment through the acquisition or development of resistance determinants [13]. MRSA have long been recognized as pathogens associated with nosocomial infections. However, lately, it has also been recognized as a major cause of community-associated infections [14]. A study by Cassini et al. estimated that around 150,000 MRSA infections occur every year resulting in over 7000 attributable deaths in European Union and the European Economic Area [15]. Besides, MRSA prevalence in Europe varies considerately among the north and south countries with south and east of the region countries reporting above-median MRSA proportions [16]. Portugal has reported a statistically significant reduction of MRSA infections between 2015 and 2019 (from 46.8% to 34.8%) but still presents higher prevalence values than other European countries [16]. MRSA of the clonal complex CC22 are among the most prevalent in Portuguese hospitals in the last decades and the epidemic MRSA (EMRSA-15) clone (ST22-IVh) accounts for more than 50% of the total isolates in hospitals [17,18,19]. This clone has also been detected among companion and wild animals in the same country [20,21]. 

Therefore, to evaluate the role of hospital wastewaters as sources of clinically relevant MRSA strains we isolated MRSA from wastewater of three Portuguese hospitals and characterized the isolates regarding the antimicrobial resistance and genetic lineages. Furthermore, since one of the hospitals had a WWTP we also isolated MRSA from water after treatment to evaluate the treatment efficacy.

## 2. Materials and Methods

### 2.1. Sample Collection

Sampling was carried out weekly over six months (October 2019 to March 2020). A total of 96 wastewater samples were collected from the Hospital Center of Trás-os-Montes and Alto Douro (CHTMAD). Twenty-four samples were collected from each hospital (Hospital of Lamego, Hospital of Chaves and Hospital of Vila Real). Since the Hospital of Vila Real was the only one that had a wastewater treatment plant on-site, we also collected 24 samples of water after treatment. The samples were collected by the authors directly in the hospital buildings using sterile 500 mL plastic bottles with sodium thiosulfate and preserved at 4–8 °C. Permission to enter the hospital and collect wastewater was previously granted and all collections were supervised by a responsible person. All samples were filtered on the same day of their collection.

### 2.2. Bacterial Isolation

Around 100 mL of hospital wastewater samples were filtered through a 0.45 µm filtration membrane. Each filter was subsequently immersed in 5 mL of Brain Heart Infusion (BHI) broth supplemented with 6.5% of NaCl and incubated at 37 °C for 24 h. Then, all samples were streaked in ORSAB (Oxacillin Resistance Screening Agar Base) agar plates with 2 mg/L of oxacillin and incubated at 37 °C for 24 to 48 h for MRSA isolation. Up to 2 colonies presenting different morphological characteristics were recovered from each plate. Species identification was accomplished using biochemical tests (catalase, DNase and coagulase tests) and by genotyping.

### 2.3. Antimicrobial Resistance Phenotype

All isolates were tested against 14 antimicrobial agents. The antimicrobial susceptibility testing was performed by the Kirby-Bauer disc diffusion method against penicillin (1 U), cefoxitin (30 μg), ciprofloxacin (5 μg), linezolid (10 μg), gentamicin (10 μg), kanamycin (30 μg), tobramycin (10 μg), erythromycin (15 μg), clindamycin (2 μg), tetracycline (30 μg), fusidic acid (10 μg), chloramphenicol (30 μg), trimethoprim/sulfamethoxazole (1.25/23.75 μg) and mupirocin (200 μg). The results were evaluated according to the EUCAST 2018 guidelines except for kanamycin which followed the guidelines of CLSI *S. aureus* strain ATCC 25923 was used as quality control in the susceptibility assays.

### 2.4. Antimicrobial Resistance and Virulence Genes

According to the antimicrobial resistance phenotypes, the respective resistance genes were investigated by PCR following conditions previously described [22]. These included the penicillin resistance gene *bla*Z, the methicillin resistance gene *mec*A, the macrolide and licosamides resistance genes *erm*A, *erm*B, *erm*C, *erm*T, *mph*C, *msr*(A/B), *lnu*A, *lnu*B, *vga*A and *vga*B, the tetracycline resistance genes *tet*K, *tet*L, *tet*M and *tet*O, the aminoglycosides resistance genes *aac*(6′)-Ie-*aph*(2′’)-Ia, *aph*(3′)-IIIa, *ant*(4′)-Ia, *str* and *ant*(6′)-Ia, the fusidic acid resistance genes *fus*A, *fus*B, *fus*C and *fus*D, the chloramphenicol resistance genes *fex*A, *fex*B, *cat*_pC194_, *cat*_pC221_, *cat*_pC223_ and *cat*A, the oxazolidinones resistance genes *cfr*, *optr*A and *poxt*A, and the trimethoprim/sulfamethoxazole resistance genes *dfr*A, *dfr*K, *dfr*D and *dfr*G. The presence of virulence genes *lukF*/*lukS*-PV, *hla*, *hlb*, *hld*, *eta*, *etb* and *tst* was detected by PCR as previously described [22]. The presence of the genes associated with the IEC system (*scn, chp*, *sak*, *sea* and *sep)* was also studied and the IEC group was ascribed accordantly [23]. Positive and negative controls used in all experiments belonged to the strain collection of the University of Trás-os-Montes and Alto Douro. 

### 2.5. Molecular Typing

All isolates were subjected to *agr* typing with the respective species-specific primers [24]. The *spa* typing was performed as described by Harmsen et al. and the *spa* types were assigned according to the public *spa* type database Ridom SpaServer (www.spaserver.ridom.de, accessed on 22 November 2021) [25]. Simple and multiple PCRs were performed to determine the type of *ccr* and *mec* complex and then staphylococcal cassette chromosome *mec* (SCC*mec*) types (I, II, III, IVa, IVb, IVc, IVd and V) were determined using multiplex PCR as previously described [26,27,28]. MLST was performed as described by Enright et al. [29]. Sequence types (STs) and clonal complexes (CCs) were assigned according to the MLST database (https://pubmlst.org/saureus/, accessed on 22 November 2021).

## 3. Results and Discussion

### 3.1. Presence of MRSA in Hospital Wastewaters

Hospital wastewaters contribute to high rates of antibiotic resistant bacteria discharged in the natural environment [30]. *S. aureus* is present in WWTP due to ineffective technological processes, yet, it has been shown that treatment significantly reduces the number of *S. aureus* in wastewaters [31]. Nevertheless, not many studies have been conducted regarding the presence of MRSA in hospital wastewaters in Europe. Although the number of MRSA strains have been decreasing in several European countries, Portugal continues to have one of the highest prevalence of MRSA in Europe at hospital level [31]. In our study, we isolated MRSA from untreated wastewater of three hospitals. Forty-five methicillin-resistant staphylococci (46.9%) were isolated from the 96 samples. From the 45 isolates, 28 (29.2%) were MRSA and the remaining 17 were coagulase-negative staphylococci. Regarding the untreated water samples, 12.5% of the 24 samples of Hospital of Lamego, 8.3% of Hospital of Chaves and 7.3% of Hospital of Vila Real were positive for MRSA. Despite the Hospital of Vila Real being the largest of the three, it was where there was a lower frequency of MRSA and the wastewater from the hospital in Lamego was the one with the highest frequency of MRSA, with half (50%) of the samples being positive for MRSA. Wastewater treatment appears to be efficient on MRSA reduction since, from the 24 treated water samples, only one was positive for MRSA which is in agreement with other studies [32,33,34]. However, Goldstein et al. argued that MRSA strains that survive to the wastewater treatment may be more likely to be multiresistant and virulent [32].

### 3.2. Antimicrobial Resistance and Virulence

All MRSA isolates (*n* = 28) were further characterized regarding the antimicrobial resistance phenotype and genotype and the presence of virulence genes. Twenty-two (78.5%) out of the 28 isolates had a multidrug-resistant profile since they were resistant to antibiotics belonging to at least 3 distinct classes (Table 1). Among them, 6 isolates exhibited resistance to three different classes of antibiotics, 8 to four classes, and 8 to five or more classes. The high prevalence of multidrug-resistant MRSA isolates may be due to selective pressure in the hospital as a result of the overuse of antibiotics [35]. All isolates were resistant to cefoxitin and penicillin and harbored the *mec*A and *bla*Z genes. The *bla*Z gene may be integrated into the chromosome or located on plasmids that may also carry genes conferring resistance to heavy metals and other antibiotic resistance genes [36]. Nowadays, it has been shown that 99% of the clinical *S. aureus* isolates are resistant to penicillin [37]. All isolates were also resistant to ciprofloxacin, which reflects the high intake of ciprofloxacin by Portuguese patients since quinolones are one of the most prescribed antibiotics in hospitals to treat soft tissue infections [18]. Furthermore, these results are in accordance with other studies conducted in patients with MRSA infections in the same hospitals [18,19,38]. Resistance to macrolides and lincosamides was detected in 22 (78.5%) out of 28 isolates and was possibly conferred by the *erm*A (*n* = 2), *erm*B (*n* = 4) or *erm*C (*n* = 20) or a combination of those genes. High frequency of resistance to macrolides and lincosamides was also reported in other studies investigating the antimicrobial resistance of MRSA strains isolated from hospital wastewater [32,39,40]. Lim et al. have shown that among the *erm* genes conferring resistance to erythromycin, only *erm*C was transmissible after transformation experiments which may explain the high prevalence of *erm*C gene in our study [41]. In general, MRSA isolates showing resistance to ciprofloxacin and erythromycin often show co-resistance to tetracycline [41]. In our study, 6 (21.4%) isolates were resistant to tetracycline and harbored the *tet*L or *tet*M genes. A similar prevalence of tetracycline was reported in a study that investigated the antimicrobial resistance of MRSA strains isolated from skin infections in the same hospital [18]. Furthermore, the isolates harbored only the *tet*L or *tet*M genes as in our study. Most tetracycline-resistant MRSA strains harbor *tet*K or *tet*M genes [42]. Nevertheless, *tet*L is also found with a moderate frequency in MRSA strains and often co-expressed with *tet*M [43]. Thirteen (46.4%) isolates showed resistance to aminoglycosides and harbored the *aph*(3′)-IIIa and *aac*(6′)-Ie-*aph*(2″)-Ia genes which is in line with the latest reports from the same hospitals indicating that treatment of MRSA infections with aminoglycosides may be no longer effective [18]. Resistance to trimethoprim-sulfamethoxazole was detected in 3 isolates harbouring the *dfr*A and/or *dfr*G genes [44,45]. Two of those isolates were also resistant to chloramphenicol mediated by the *cat*_pC221_ gene [46]. Although chloramphenicol resistance is still low in MRSA isolates from humans in Portugal it has been reported at a moderate frequency in swine [18,47,48,49]. The MRSA strain VS2939 was the only one isolated from treated wastewater and it did not present a multidrug-resistance profile [32].

The *scn* gene is a marker of the immune evasion cluster (IEC) system as is a common gene in all IEC groups. The *chp*, *sak*, *sea* and *sep* genes were tested in all isolates positive for *scn* and the IEC group was ascribed accordantly. There are seven different types of IEC (from type A to type G) depending on the combination of the genes *scn*, *chp*, *sak*, *sea* and *sep* which have different functions for survival of *S. aureus* [23]. The presence of *scn* gene, screened in all isolates, was detected in 24 (85.7%). The positive isolates were further screened for the presence of the other IEC genes. Twenty-three isolates carried the *chp* and *sak* genes and were ascribed to IEC type B while only one isolate carried the *sak* and *sea* genes and was ascribed to type D. The IEC system plays an important role in human colonization, and it has been reported that type B is the predominant variant in isolated from human infections [23]. In Portugal, the presence of IEC system has not been frequently studied in clinical isolates but in a previous study carried out by our research team on MRSA skin infection isolates, we found that the most prevalent was also type B [18]. Regarding the presence of other virulence genes, 26 (92.9%), 25 (89.3%) and 10 (35.7%) isolates carried the *hlb*, *hld* and *tst* genes. None of the isolates carried the virulence genes *hla*, *eta*, *etb* and *lukF*/*lukS*-PV. The gene *tst* encodes the staphylococcal toxic shock syndrome toxin and a low frequency of this gene has been reported among MRSA strains isolated from human patients in Portugal and some other European countries, such as Greece and the Czech Republic, while in other countries this prevalence is higher [19,50,51,52]. These differences may be related to specific MRSA clones.

### 3.3. Molecular Typing

All isolates were genotyped based on MLST, SCC*mec*, *spa* and *agr*-typing. Of the 28 MRSA isolates, 26 were ascribed to ST22, one to ST8 and the last one to STThe ST22 isolates were divided into five *spa*-types: t747 (*n* = 20), t6966 (*n* = 2), t020 (*n* = 2), t1302 and tAll ST22 isolates belonged to SCC*mec* type IV and *agr* type I. The ST22-MRSA-IV is known as the epidemic clone EMRSA-15 and is a hospital-associated pathogen. This clone is typically ciprofloxacin and erythromycin resistant which is in line with our results [53]. EMRSA-15 emerged in the UK three decades ago, has spread globally and has been reported in several countries worldwide [54,55,56,57]. EMRSA-15 is also the predominant clone circulating in Portuguese hospitals and, therefore, it was no surprise that this clone was also the most frequent in hospital wastewater in Portugal [18,58]. Furthermore, the genetic and phenotypic traits of the EMRSA-15 strains isolated in this study are similar to the characteristics of the EMRSA-15 strains isolated from infections of patients hospitalized in the same hospitals [18,19]. These isolates also showed high frequency of resistance to penicillin (encoded by *bla*Z gene) and to erythromycin (mainly encoded by *erm*C). The *spa*-type t747 is highly associated with ST22-MRSA. In fact, several Portuguese studies have reported the ST22-MRSA-t747 clone in human infections, wild animals and dogs [18,19,20,21,59]. One isolate belonged to ST8, *spa*-type t008, SCC*mec* IV and *agr* I. The ST8-MRSA-IV clone is a dominant community-associated MRSA clone also known as USAThis is the dominant clone causing infections in the United States and Europe, particularly, skin and soft tissue infections [60]. However, USA300 clones are usually multidrug-resistant, carry the arginine catabolic mobile element (ACME) and are positive for genes encoding PVL and in our study, none of the isolates was positive for PVL encoding gene [60,61]. Although the prevalence of the USA300 clone in Portugal is low, this clone has been reported in many European countries [19,62,63,64]. Finally, one MRSA strain isolated in our study belonged to ST105, *spa*-type t10682, SCC*mec* II and *agr* II. Most clones found in hospital wastewaters were ascribed to SCC*mec* type IV. Börjesson et al. has found a high abundance of MRSA SCC*mec* type IV in wastewaters and suggested that strains belonging to this SCC*mec* type may have superior survival characteristics due to the lower energy cost of SCC*mec* IV carriage [65]. 

## 4. Conclusions

MRSA strains were isolated from hospital wastewaters, most of which had multidrug-resistance pheno- and genotypes, suggesting the importance of hospital effluents as sources of bacteria clinically relevant to the environment. Indeed, all MRSA isolates were ascribed to recognized epidemic clones. Moreover, it is suggested that these effluents may contribute to enriching the gene pool for antibiotic resistant bacteria in the environment. Generally, hospital effluents are discharged directly into public sanitation networks, without any prior treatment. The wastewater treatment process used in one of the hospitals seems to contribute to reducing the burden of the effluent, at least in what refers to *S. aureus*. Therefore, our results reinforce the importance of implementing wastewater treatment systems in hospitals allowing a decrease in the survival of MRSA strains and their dissemination.

## Figures and Tables

**Table 1:** Description and genetic characteristics of MRSA strains isolated from hospital wastewaters in Portugal.

Isolate	Hospital (Treated/Untreated Wastewater)	Antimicrobial Resistance		Virulence Factors			Molecular Typing		
		Phenotype	Genotype	IEC Type	Other Genes	ST (CC)	*spa*	SCC*mec*	*agr*
**VS2932**	Vila Real (Untreated)	BEN,CXI, CIP, ERY	*mec*A, *bla*Z, *erm*A	B	*hld*	105	t10682	II	II
**VS2933**	Vila Real (Untreated)	BEN, CXI, CIP, GEN, ERY, CLI, FUS	*mec*A, *bla*Z, *aph*(3′)-IIIa, *erm*C	B	*tst*	22 (22)	t747	IV	I
**VS2934**	Vila Real (Untreated)	BEN, CXI, CIP, GEN, ERY	*mec*A, *bla*Z, *aph*(3′)-IIIa, *erm*C	B	*hld*	22 (22)	t747	IV	I
**VS2935**	Vila Real (Untreated)	BEN, CXI, CIP, GEN, TOB, ERY, CLI, FUS	*mec*A, *bla*Z, *aph*(3′)-IIIa, *erm*C	B	*hld, tst*	22 (22)	t6966	IV	I
**VS2936**	Vila Real (Untreated)	BEN, CXI, CIP, GEN, KAN, TOB, ERY, CLI, TET, CHL, FUS, TRS	*mec*A, *bla*Z, *aph*(3′)-IIIa, *erm*C, *tet*L, cat_pC221_, *dfr*A	B	*hld*	22 (22)	t747	IV	I
**VS2937**	Vila Real (Untreated)	BEN, CXI, CIP	*mec*A, *bla*Z	B	*hld*	22 (22)	t020	IV	I
**VS2938**	Vila Real (Untreated)	BEN, CXI, CIP, ERY	*mec*A, *bla*Z, *erm*C	B	*hld*	22 (22)	t747	IV	I
**VS2939**	Vila Real (Treated)	BEN, CXI, CIP, ERY	*mec*A, *bla*Z, *erm*C	-	*hld*	22 (22)	t020	IV	I
**VS2940**	Chaves (Untreated)	BEN, CXI, CIP, GEN, ERY	*mec*A, *bla*Z, *aac*(6′)-Ie-*aph*(2′’)-Ia, *aph*(3′)-IIIa, *erm*B, *erm*C	B	*hld*	22 (22)	t747	IV	I
**VS2941**	Chaves (Untreated)	BEN, CXI, CIP, LNZ, GEN	*mec*A, *bla*Z, *aph*(3′)-IIIa, *tet*L	-	*hld, tst*	22 (22)	t19963	IV	I
**VS2942**	Chaves (Untreated)	BEN, CXI, CIP, GEN, KAN, ERY, CLI, FUS	*mec*A, *bla*Z, *aph*(3′)-IIIa, *erm*C	B	*hld*	22 (22)	t747	IVc	I
**VS2943**	Chaves (Untreated)	BEN, CXI, CIP, ERY	*mec*A, *bla*Z, *erm*C	B	*hld, tst*	22 (22)	t747	IVc	I
**VS2944**	Chaves (Untreated)	BEN, CXI, CIP, ERY, CLI, FUS	*mec*A, *bla*Z, *erm*C	*-*	*hlb, hld, tst*	22 (22)	t747	IVc	I
**VS2945**	Chaves (Untreated)	BEN, CXI, CIP, ERY, TET	*mec*A, *bla*Z, *erm*C, *tet*M	B	*hld*	22 (22)	t747	IV	I
**VS2946**	Chaves (Untreated)	BEN, CXI, CIP, GEN, KAN, TOB, ERY, CLI, TET, FUS, TRS	*mec*A, *bla*Z, *aac*(6′)-Ie-*aph*(2′’)-Ia, *aph*(3′)-IIIa, *erm*B, *erm*C, *tet*L, *dfr*G	-	*hlb, hld*	22 (22)	t6966	IV	I
**VS2947**	Chaves (Untreated)	BEN, CXI, CIP	*mec*A, *bla*Z	B	*hld*	22 (22)	t747	IV	I
**VS2948**	Lamego (Untreated)	BEN, CXI, CIP, ERY	*mec*A, *bla*Z, *erm*C	B	*hld*	22 (22)	t747	IVc	I
**VS2949**	Lamego (Untreated)	BEN, CXI, CIP	*mec*A, *bla*Z	B	*hld*	22 (22)	t747	IV	I
**VS2950**	Lamego (Untreated)	BEN, CXI, CIP, GEN, ERY	*mec*A, *bla*Z, *aph*(3′)-IIIa, *erm*C	B	*hld*	22 (22)	t747	IV	I
**VS2951**	Lamego (Untreated)	BEN, CXI, CIP, GEN, TOB, ERY	*mec*A, *bla*Z, *aac*(6′)-Ie-*aph*(2′’)-Ia, *aph*(3′)-IIIa, *erm*B, *erm*C	B	*hld*	22 (22)	t1302	IVc	I
**VS2952**	Lamego (Untreated)	BEN, CXI, CIP, GEN, KAN, TOB, ERY, CLI, TET, CHL, FUS, TRS	*mec*A, *bla*Z, *aph*(3′)-IIIa, *erm*B, *erm*C, *tet*L, *dfr*G, *dfr*A,cat_pC221_	B	*hld*	22 (22)	t747	IVc	I
**VS2953**	Lamego (Untreated)	BEN, CXI, CIP	*mec*A, *bla*Z	B	*hld, tst*	22 (22)	t747	IVc	I
**VS2954**	Lamego (Untreated)	BEN, CXI, CIP, GEN, ERY	*mec*A, *bla*Z, *aph*(3′)-IIIa, *erm*C	B	*hld, tst*	22 (22)	t747	IVc	I
**VS2955**	Lamego (Untreated)	BEN, CXI, CIP, ERY	*mec*A, *bla*Z, *erm*C	B	*hld*	22 (22)	t747	IVc	I
**VS2956**	Lamego (Untreated)	BEN, CXI, CIP, ERY, FUS	*mec*A, *bla*Z, *erm*C	B	*tst*	22 (22)	t747	IV	I
**VS2957**	Lamego (Untreated)	BEN, CXI, CIP, GEN, KAN, ERY, CLI, FUS	*mec*A, *bla*Z, *aac*(6′)-Ie-*aph*(2′’)-Ia, *aph*(3′)-IIIa, *erm*C, *erm*A	D	*hld, tst*	8	t008	IV	I
**VS2958**	Lamego (Untreated)	BEN, CXI, CIP	*mec*A, *bla*Z	B	*hld*	22 (22)	t747	IV	I
**VS2959**	Lamego (Untreated)	BEN, CXI, CIP	*mec*A, *bla*Z	B	*tst*	22 (22)	t747	IV	I

Abbreviations: CXI: cefoxitin; BEN: penicillin; ERY: erythromycin; CLI: clindamycin; GEN: gentamicin; KAN: kanamycin; TOB: tobramycin; CIP: ciprofloxacin; FUS: fusidic acid; TET: tetracycline; CHL: chloramphenicol; TRS: trimethoprim-sulfamethoxazole; MLST: multilocus sequence typing; ST: sequence type; CC: clonal complex; SCC*mec*: staphylococcal cassette chromosome; IEC: immune evasion cluster.
